# An Important Role of the SDF-1/CXCR4 Axis in Chronic Skin Inflammation

**DOI:** 10.1371/journal.pone.0093665

**Published:** 2014-04-02

**Authors:** Silvana Zgraggen, Reto Huggenberger, Katrin Kerl, Michael Detmar

**Affiliations:** 1 Institute of Pharmaceutical Sciences, Swiss Federal Institute of Technology, ETH Zurich, Zurich, Switzerland; 2 Department of Dermatology, University Hospital Zurich, Zurich, Switzerland; University of Bari Medical School, Italy

## Abstract

Inflammatory angiogenesis and vascular remodeling play key roles in the chronic inflammatory skin disease psoriasis, but little is known about the molecular mediators of vascular activation. Based on the reported elevated mRNA levels of the angiogenic chemokine stromal cell-derived factor-1 (SDF-1) and its receptor CXCR4 in psoriasis, we investigated the relevance of the SDF-1/CXCR4 axis in two experimental models of chronic psoriasis-like skin inflammation. The cutaneous expression of both SDF-1 and CXCR4 was upregulated in the inflamed skin of K14-VEGF-A transgenic mice and in imiquimod-induced skin inflammation, with expression of CXCR4 by blood vessels and macrophages. Treatment with the CXCR4 antagonist AMD3100 potently inhibited skin inflammation in both models, associated with reduced inflammatory angiogenesis and inflammatory cell accumulation, including dermal CD4^+^ cells and intraepidermal CD8^+^ T cells. Similar anti-inflammatory effects were seen after treatment with a neutralizing anti-SDF-1 antibody. In vitro, inhibition of CXCR4 blocked SDF-1-induced chemotaxis of CD11b^+^ splenocytes, in agreement with the reduced number of macrophages after in vivo CXCR4 blockade. Our results reveal an important role of the SDF-1/CXCR4 axis in skin inflammation and inflammatory angiogenesis, and they indicate that inhibition of the SDF-1/CXCR4 axis might serve as a novel therapeutic strategy for chronic inflammatory skin diseases.

## Introduction

Psoriasis is a chronic inflammatory skin disease that affects 2% to 3% of the population worldwide [1]. The major features of psoriatic skin lesions are the thickening of the epidermis, associated with enhanced proliferation and aberrant terminal differentiation of epidermal keratinocytes, and the accumulation of inflammatory leukocytes, in particular dendritic cells and T cells [2]. There is a preferential accumulation of CD4^+^ cells in the dermis and of CD8^+^ cells in the epidermis of lesional psoriatic skin [2], and there are also increased numbers of macrophages whose function in the disease process is not fully understood [3].

A third – and often overlooked - hallmark of psoriatic skin is represented by the pronounced inflammatory angiogenesis, leading to vascular remodeling [4]. This vascular activation is similar to the blood vessel changes observed in several other chronic inflammatory diseases, including rheumatoid arthritis, inflammatory bowel disease and chronic airway inflammation [5]–[7]. Vascular endothelial growth factor (VEGF)-A has been identified as the major angiogenesis factor in psoriatic skin since its expression is strongly upregulated in lesional epidermis [5], and the plasma levels of VEGF-A are elevated in psoriasis patients and correlate with disease severity [8]. Moreover, targeted chronic overexpression of VEGF-A in the epidermis of mice results in the spontaneous development of a chronic inflammatory skin disease that shares many features of human psoriasis [9], [10], and inhibition of VEGF-A signaling inhibited inflammation in different murine models of psoriasis [9]–[12]. Together, these results indicate an important role of VEGF-A and angiogenesis in disease maintenance and progression, but additional factors that target the vascular system are likely involved.

Several pro-inflammatory chemokines have been reported to also exhibit angiogenic properties [13], [14]. In particular, stromal cell-derived factor-1 (SDF-1, also known as CXCL12) has been found to have angiogenic activity in different in vitro and in vivo systems [15], [16], and its primary receptor CXCR4 is expressed by endothelial cells as well as monocytes and lymphocytes [17], [18]. SDF-1 is expressed in several tissues including the skin and the bone marrow [19], and the SDF-1/CXCR4 axis is involved in a range of physiological processes such as embryonic development and stem cell motility [20]. Since VEGF-A upregulates CXCR4 in cultured human endothelial cells [16], and since elevated mRNA levels of both SDF-1 and CXCR4, as well as elevated SDF-1 protein levels, have been found in lesional psoriatic skin [21]–[23], we hypothesized that the SDF-1/CXCR4 axis might play a potential role in the pathogenesis of chronic inflammatory skin diseases including psoriasis.

Therefore, we first investigated the expression of CXCR4 in human psoriasis and of both SDF-1 and CXCR4 in two established experimental mouse models of chronic psoriasis-like skin inflammation - the keratin 14 (K14)–VEGF-A transgenic mouse model of induced chronic cutaneous inflammation [9], and the imiquimod-induced psoriasis-like skin inflammation mouse model [24], [25]. Next, we investigated the potential anti-inflammatory effects of blockade of CXCR4 signaling by systemic treatment with AMD3100, a specific CXCR4 antagonist [26], in both inflammation models, and we also studied the effects of a neutralizing anti-SDF-1 antibody. Our results reveal an important role of the SDF-1/CXCR4 axis in skin inflammation and inflammatory angiogenesis, and they indicate that inhibition of the SDF-1/CXCR4 axis might serve as a novel treatment strategy for chronic inflammatory skin diseases.

## Materials and Methods

### Ethics Statement

The collection of specimens from clinically indicated excisions for this study was explicitly approved by the institutional review board (Kantonale Ethikkommission Zürich). Informed consent (both written and verbal) was obtained from patients for the use of their skin samples in this research project.

### Mice and treatments

All mice used in this study were bred and housed in the animal facility of ETH Zurich. Experiments were performed in accordance with animal protocol 149/2008 and 117/2011 approved by the local veterinary authorities (Kantonales Veterinäramt Zürich). K14-VEGF-A transgenic mice have been described previously [10], [27]. To induce a psoriasis-like skin inflammation in the ear skin [9] of these mice, a 2% oxazolone solution (4-ethoxymethylene-2 phenyl-2-oxazoline-5-one; Sigma-Aldrich) in aceton/olive oil (4∶1 vol/vol) was applied topically to the shaved abdomen (50 μl) and to each paw (5 μl) of 8-weeks old females. Five days after sensitization (day 0), both ears were challenged by topical application of 10 μl oxazolone (1%) on each side. Ear thickness was measured before challenge and repeatedly after challenge using calipers. Starting on day 7, 10 mg/kg of AMD3100 octahydrochloride hydrate (1,1′-[1,4-Phenylenebis(methylene)]bis-1,4,8,11-tetraazacyclotetradecaneoctahydrochloride, Sigma-Aldrich) or PBS (vehicle) was administered subcutaneously into the loose skin over the neck of the mice for 14 days every 12 hours (n = 10 per group). The AMD3100 dose was chosen based on previous studies [28].Additionally, inflamed K14-VEGF-A mice were treated with 50 μg of a neutralizing mouse anti-mouse SDF-1 monoclonal antibody (MAB310; R&D Systems) or control mouse IgG (MAB002; R&D Systems), administered i.v. from day 7 to day 21, every second day (n = 7 per group). On day 21, mice were injected with 300 μl PBS containing 40 mM BrdU (Sigma-Aldrich) and killed 2.5 h after the injection. Ears and ear draining lymph nodes (LN) were embedded in optimal cutting temperature (OCT) compound (Sakura Finetek) or used for FACS analysis (see Materials and Methods S1 in File S1) and RNA isolation.

In a second model of psoriasis, C57BL/6 wild-type mice (n = 5) received daily topical applications of imiquimod, using a commercially available cream (5%) (Aldara: 3 M Pharmaceuticals), on the ear skin for 8 consecutive days AMD3100 treatment was started 12 h before the first imiquimod treatment and repeated every 12 hours as described in the section above. Ear thickness was measured every day, starting before the first AMD3100 treatment. Ears and ear draining LN were embedded in OCT or used for RNA isolation. For all mice, the weight of the ear draining LN was determined.

### Immunofluorescence

Tissues were embedded in OCT, frozen on liquid nitrogen, and 7 μm cryostat sections were cut. Specimens were placed on glass slides, air dried and fixed with aceton for 2 min at −20°C. After rehydration with 80% methanol at 4°C, the specimens were washed in PBS and then in PBS with 5% donkey serum and 1% bovine serum albumin, followed by incubation with the respective primary antibodies. Standard H&E and immunofluorescence stainings were performed as described previously [9], [29], using the following antibodies: anti-mouse LYVE-1 (AngioBio), biotin anti-MECA-32 (BD Biosciences), anti-human von Willebrand factor (Dako), anti-mouse podoplanin (clone 8.1.1, Developmental Studies Hybridoma Bank, University of Iowa), anti-mouse keratin 6, anti-loricrin (Covance Research Products), anti-BrdU-Alexa Fluor 594 (Invitrogen), anti-mouse F4/80 (AbD Serotec), anti-mouse CD68 (Abcam), anti-mouse CD8 (BD Biosciences) and anti-mouse CXCR4 (R&D Systems). Alexa Fluor488-, Alexa Fluor594- and Alexa Fluor647-coupled secondary antibodies and Hoechst 33342 were purchased from Invitrogen.

### Immunohistochemistry

Immunohistochemistry was performed on 2 μm paraffin sections of normal human skin (n = 8) and of lesional psoriatic skin (n = 8), using an anti-human CXCR4 antibody (R&D Systems) or control mouse IgG (Sigma) followed by a biotinylated anti-mouse secondary antibody (Vector). The Vectastain ABC and the AEC (3-amino-9-ethylcarbazole) substrate kits (Vector) were used for chromogenic detection.

### Computer-assisted morphometric analyses

Double immunofluorescence stains of ear sections for MECA-32 and LYVE-1 were examined, and computer-assisted analysis of digital images were performed using an in-house designed ImageJ macro. The average number of LYVE-1^+^ lymphatic vessels and MECA-32^+^ blood vessels per millimeter epidermal basement membrane and the average size of vessels were determined in the area between cartilage and epidermis of one ear half. The results are expressed as vessel number per millimeter of epidermal basement membrane (excluding follicular structures) [29] and not as vessel number per area because the formation of inflammatory edema (increase in area) would confound the vessel number if it were calculated per area. To quantify CD8^+^ and BrdU^+^ cell numbers per millimeter of epidermal basement membrane, images of four individual fields of view were acquired per sample. Quantification of F4/80^+^, CD68^+^ and CXCR4^+^ areas was done by measuring the immunostained areas using gray-scale detection with a fixed threshold in ImageJ (National Institutes of Health, Bethesda) and was expressed as positive stained area per region of interest (one ear half).

### Image acquisition

Stainings were examined using an Axioskop2 mot plus microscope (Carl Zeiss, Inc.), equipped with an AxioCamMRc camera and a Plan-APOCHROMAT 10x/0.45 NA objective (Carl Zeiss, Inc.) Images were acquired using Axio Vision Version 4.4 software (Carl Zeiss). Confocal images were acquired with a LSM710 FCS confocal microscope and ZEN software (Zeiss, Jena, Germany) and were processed with IMARIS software (Verstion 7.1.1, Bitplane AG, Zürich, Switzerland).

### Quantitative real-time RT-PCR

Total cellular RNA was isolated from mouse ears using a TissueLyser, stainless steel beads, and the RNeasy Mini kit (QIAGEN). 1 μg RNA was used to generate cDNA using the High-Capacity cDNA Reverse Transcription kit (Applied Biosystems). The expression of mouse CXCR4, SDF-1, CXCR7, CXCL3, CCL20, S100a7/8 was investigated by SYBR Green real-time reverse-transcribed polymerase chain reaction (RT-PCR) using the AB7900 HT Fast Real-Time PCR System (Applied Biosystems) and was quantified using the 2^−ΔΔCt^ method [30]. The primers for mouse CXCR4, SDF-1, CXCR7, CXCL3, CCL20, S100a7/a8 were custom-made oligonucleotide primers (Microsynth, Switzerland). Each reaction was run with β-actin as a reference gene, and all data were normalized based on the expression levels of β-actin; n = 5 per group.

### Isolation of CD11b^+^ splenocytes and transwell migration assays

Splenic CD11b^+^ cells were isolated from wildtype FVB mice by positive selection using mouse CD11b MicroBeads (Miltenyi Biotech). CXCR4 expression of CD11b^+^ splenocytes was analyzed by flow cytometry. A single cell suspension of CD11b^+^ splenocytes was stained at 4°C for 30 minutes with PE-labeled rat anti-mouse CXCR4 antibody (eBioscience) or isotype control antibody (BioLegend). FACS was performed on a BD FACSCanto (BD Biosciences) using FACSDiva software. Data were analyzed with FlowJo software. Transwell migration assays were performed using polycarbonate 5 μm pore size-membrane 24-well plates (Corning Life Sciences). When AMD3100 was tested, cells were pre-incubated for 30 minutes with the compound. 4×10^5^ CD11b^+^ splenocytes were seeded in 0.2% BSA containing medium (DMEM, Invitrogen) into the upper part of the transwell insert, which was coated with 10 μg/ml fibronectin (Millipore) on the lower side and blocked with 100 μg/ml BSA as described [31]. Cells were allowed to migrate to the lower chamber containing either 0.2% BSA, additional recombinant SDF-1 (10, 100 or 500 ng/ml; Peprotech) alone or in combination with anti-SDF antibody or control IgG antibodies (10 μg/ml) for 3.5 h. After the incubation time, non-migrated cells were mechanically removed and the migrated cells were stained with Hoechst. Five images per insert were taken and nuclei from 3 wells per condition were counted using ImageJ.

### Detection of endothelial CXCR4 expression by FACS

Primary human dermal blood vascular endothelial cells (BEC) and lymphatic endothelial cells (LEC) were isolated from neonatal human foreskins as previously described [32]. BECs were cultured in endothelial basal medium (EBM; Lonza), supplemented with 20% fetal bovine serum (FBS; Invitrogen), 2 mM L-glutamine, antibiotic-antimycotic solution (Invitrogen), and 10 μg/ml hydrocortisone (Sigma). LECs were cultured in the same medium with additional N6,2-O-dibutyryladenosine-3′,5′-cyclic monophosphate (25 μg/mL; Sigma). Cells were used at passage 6. For FACS analyses, BECs and LECs were stained with PE-labeled anti-human CXCR4 (eBioscience) or isotype control antibody (BioLegend).

### Statistical analyses

Statistical analyses were performed using Prism version 4.0 (GraphPad Software, Inc). Data are shown as mean ±SD or ±SEM as indicated and were analyzed with a 2-tailed, unpaired Student's *t*-test whentwo groups were compared, a one-way ANOVA with Bonferroni post-test when more than two groups were compared, and a two-way Anova with Bonferroni post-test for repeated measurements. Homogeneity of variances was assessed using Levene's test, and normalized distribution was assessed using Q-Q-plots. Differences were considered statistically significant when *P*≤0.05.

## Results

### Chronic skin inflammation leads to up-regulation of CXCR4 and SDF-1 and to accumulation of CXCR4^+^ cells

**Figure 1:**
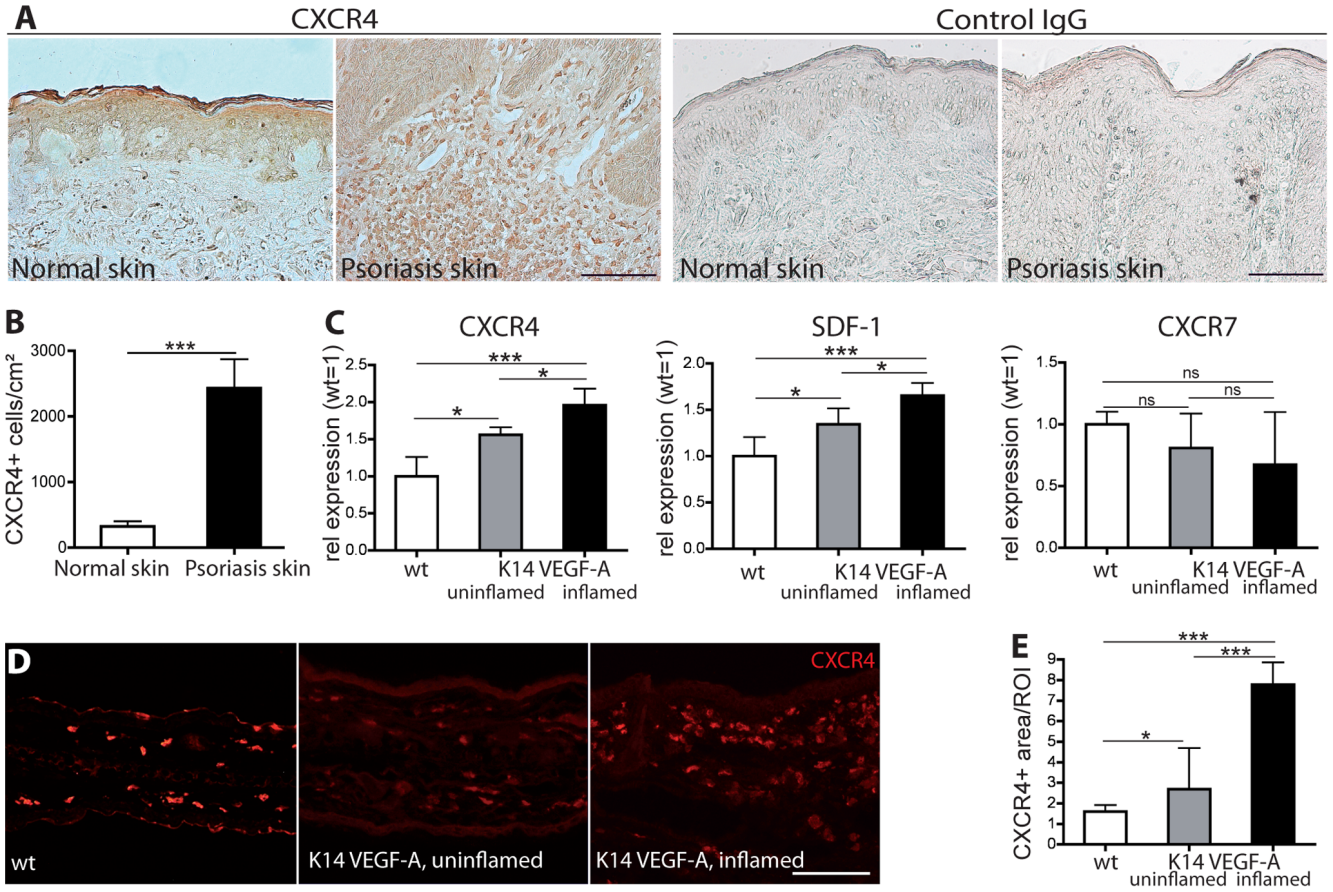
Increased number of CXCR4^+^ cells in human psoriatic skin and up-regulation of CXCR4 and SDF-1 in experimental chronic skin inflammation. (A–B) Immunohistochemistry revealed, that in normal human skin, CXCR4 is mainly expressed in the epidermis. In psoriatic skin, CXCR4 is also expressed on a big number of infiltrating cells. Quantitative image analysis showed that significantly more CXCR4^+^ cells were present in the dermis of psoriatic skin lesions (n = 8) than in normal human skin (n = 8). Staining with isotype matched control IgG confirmed the specificity of the CXCR4 staining. Scale bar represents 100 μm. ^***^P<0.001. (C) Real-time RT-PCR analysis of RNA obtained from whole ear skin extracts of inflamed K14-VEGF-A transgenic mice (day 21 after oxazolone challenge), untreated K14-VEGF-A transgenic, and wild-type mice (n = 5 per group). The expression of CXCR4 and SDF-1 was significantly up-regulated in uninflamed K14-VEGF-A transgenic mouse skin compared to wild-type mice and was further increased in the inflamed skin of K14-VEGF-A transgenic mice. The expression of CXCR7 was slightly lower in the skin of K14-VEGF-A transgenic mice. (D–E) Immunofluorescence stains for CXCR4 revealed that inflamed K14-VEGF-A transgenic mice have a significantly increased CXCR4^+^ tissue area as compared with uninflamed K14-VEGF-A transgenic mice and wild-type mice. Scale bar represents 100 μm. Data represent mean ± SD. ^*^P<0.05; ^***^P<0.001. ns, not significant.

We first investigated whether CXCR4 might play a role in human psoriasis. To this end, we performed immunohistochemical stains for CXCR4 on sections of normal human skin and of psoriatic skin lesions. In normal human skin, the CXCR4 expression was largely restricted to epidermal keratinocytes (Fig. 1A). In comparison, in psoriatic lesional skin, CXCR4 was also expressed on a large number of infiltrating single cells, mostly represented by macrophages. No staining was observed with isotype matched control IgG. Additionally, CXCR4 expression was also seen on some vascular endothelial structures. The number of CXCR4^+^ cells in the dermis, was significantly increased (+652%, P<0.001) in psoriatic skin compared to normal human skin (Fig. 1B). Next, we investigated whether the SDF-1/CXCR4 axis might also be involved in the experimental skin inflammation model of hemizygous K14-VEGF-A transgenic mice. These mice develop a chronic, psoriasis-like skin inflammation after sensitization and challenge with the contact sensitizer oxazolone [9]. Real-time RT-PCR analyses revealed a significantly increased expression of CXCR4 (2.0-fold, P<0.001) and SDF-1 (1.7-fold, P<0.001) in the inflamed (21 days after oxazolone challenge) ear skin of K14-VEGF-A transgenic mice as compared to the uninflamed skin of wild-type mice (Fig. 1C). There was also a slight upregulation of CXCR4 and SDF-1 in the non-inflamed skin of the transgenic mice compared to wild-type mice, likely due to the reported proinflammatory status of these mice [27].

In contrast, no significant differences in the expression of CXCR7, another SDF-1 receptor, were found between wild-type skin, uninflamed and inflamed skin of K14-VEGF-A transgenic mice, although levels were slightly lower under inflamed conditions (Fig. 1C). Immunofluorescence stainings showed that in the inflamed skin of K14-VEGF-A transgenic mice, more cells expressed CXCR4 than in uninflamed skin of K14-VEGF-A transgenic and of wild-type mice (Fig. 1D). Quantitative image analyses revealed that the percentage of tissue area that stained positively for CXCR4 was significantly increased in the inflamed ear skin of K14-VEGF-A transgenic mice as compared with wildtype mice (384%, P<0.001) and uninflamed K14-VEGF-A transgenic mice (189%, P<0.001) (Fig. 1E).

### Blockade of CXCR4 reduces chronic skin inflammation and inflammatory angiogenesis

**Figure 2:**
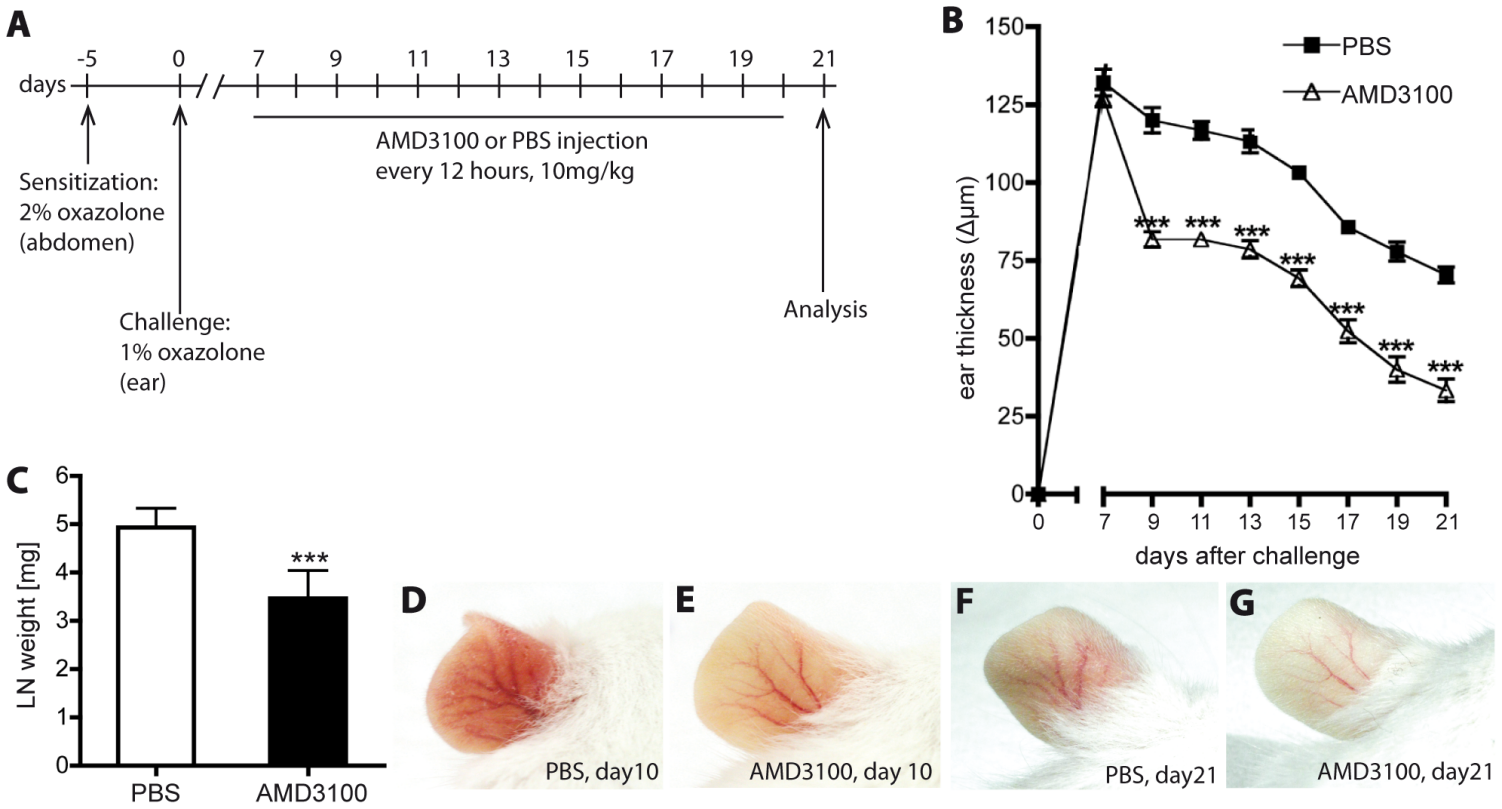
Inhibition of CXCR4 reduces edema formation in chronic skin inflammation. (A) Hemizygous K14-VEGF-A transgenic mice (n = 20) were sensitized with 2% oxazolone on day -5 and challenged on day 0 by topical application of 1% oxazolone on the ears. Starting on day 7, mice received s.c injections of AMD3100 (CXCR4-antagonist) or PBS (vehicle) (n = 10 per group) every 12 hours. Two independent experiments were performed. (B) Treatment with AMD3100 (△) reduced ear swelling, as compared with PBS-treated controls (□) Data represent mean±SEM. (C) Reduced weight of ear draining LN after 14 days of AMD3100 treatment (day 21), as compared with PBS-injected mice. Data represent mean±SD. ^***^P<0.001. (D-G) AMD3100-treated animals showed reduced ear redness on day 10 (E) and day 21 (G) as compared to PBS-treated animals (D,F).

We next investigated the effects of AMD3100, a selective CXCR4 antagonist [26], on the inflammatory response. K14-VEGF-A transgenic mice were sensitized with oxazolone (day -5) and challenged 5 days later by topical application of oxazolone on both sides of the ears. 7 days after challenge, the mice received s.c. injections of AMD3100 or PBS every 12 hours. On day 21, the mice were analyzed, 12 hours after receiving the last injection (Fig. 2A). AMD3100 treatment significantly reduced edema formation in the ears already after 2 days of treatment (day 9; −31.85%; P<0.001) as compared with control PBS-injected mice (Fig. 2B). The anti-inflammatory effect of AMD3100 was reflected by the significantly reduced weight of the ear draining LN after 14 days of AMD3100 treatment (−29.76%; P<0.001; Fig. 2C). Accordingly, ear redness was also reduced after 3 (day 10) and 14 days (day 21) of AMD3100 treatment (Fig.2 D-G).

**Figure 3:**
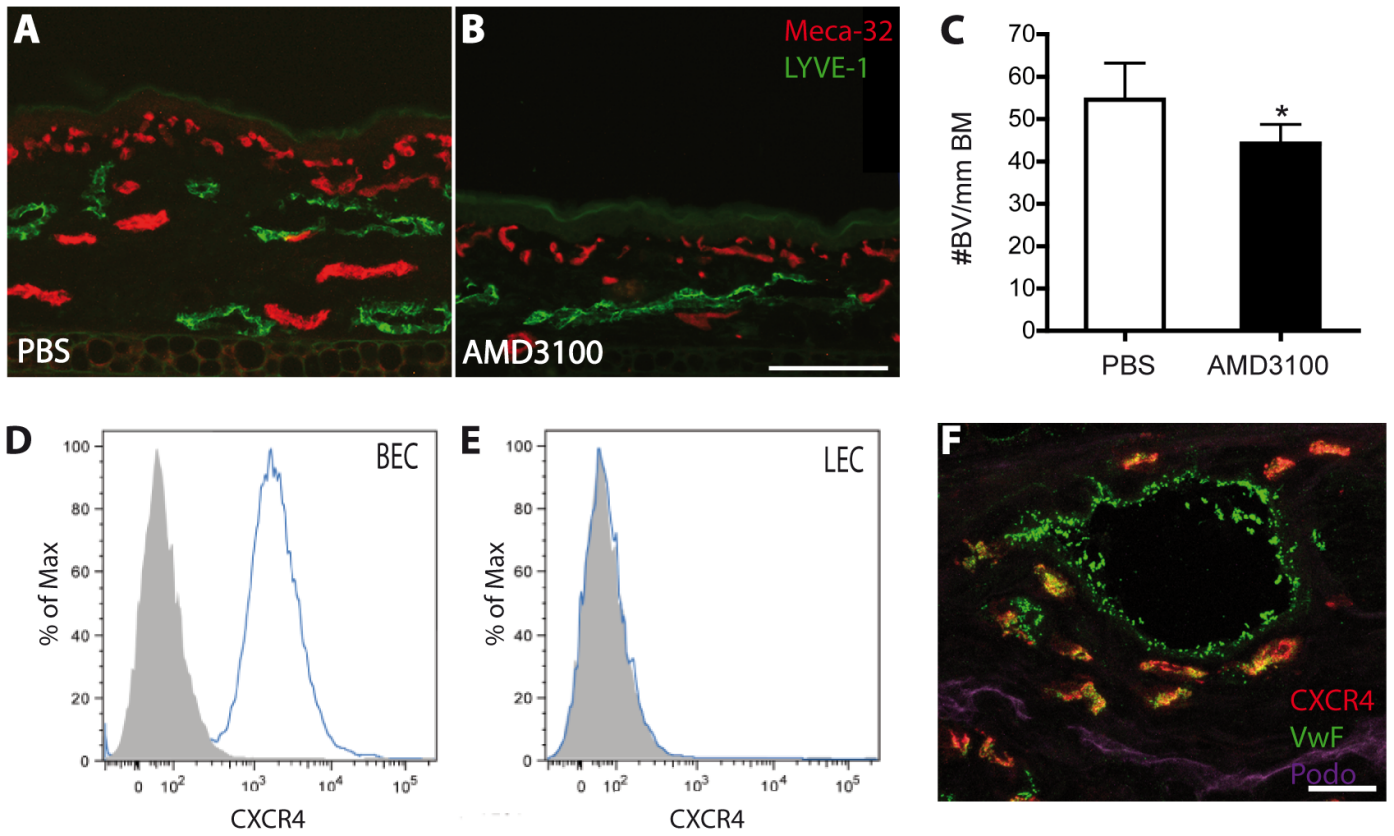
Inflammatory angiogenesis is reduced by inhibition of CXCR4. (A–B) Representative fluorescent images of MECA-32^+^ blood vessels (red) and LYVE-1^+^ lymphatic vessels (green) in the inflamed ear skin of PBS (A) and AMD3100-treated (B) K14-VEGF-A transgenic mice. One ear half is shown. Scale bar represents 100 μm. (C) Quantitative image analysis of MECA-32^+^ blood vessels (BV) revealed significantly reduced numbers of blood vessels per millimeter basement membrane (BM) in the inflamed ear skin of AMD3100-treated mice, as compared with PBS-treated control mice. (D–E) FACS analysis of CXCR4 expression by dermal blood vascular endothelial cells (BEC; D) and lymphatic endothelial cells (LEC; E) revealed that CXCR4 is expressed by BEC but not by LEC in vitro. (F) Immunofluorescence staining for CXCR4 (red), von Willebrand factor (VwF, green) and Podoplanin (Podo, purple) of inflamed ear skin (confocal image) demonstrates specific CXCR4 expression by blood vessels (BV) but not lymphatic vessels (LV). Scale bar represents 20 μm. Data represent mean ±SD. ^*^P<0.05.

To study potential changes in cutaneous vascularization, we next performed immunostains for the blood vessel marker MECA-32 and for the lymphatic vessel marker LYVE-1 (Fig. 3A-B). The number of blood vessels was significantly lower in the skin of AMD3100-treated mice than in control mice (-19.94%; p<0.05; Fig. 3C). In contrast, there was no significant change in blood vessel size, lymphatic vessel number or lymphatic vessel size (data not shown). These results are in agreement with our in vitro findings, obtained by FACS analysis of cultured human cells, that CXCR4 is a blood vessel endothelial cell specific gene that is not expressed by lymphatic endothelial cells (Fig. 3D–E). Immunofluorescence stains of inflamed ear skin revealed that CXCR4 was indeed expressed by von Willebrand factor-positive blood vessels but not by podoplanin-positive lymphatic vessels (Fig. 3F).

#### Neutralization of SDF-1 reduces chronic skin inflammation and inhibits leukocyte migration towards SDF-1 in vitro to a similar extent as AMD3100

**Figure 4:**
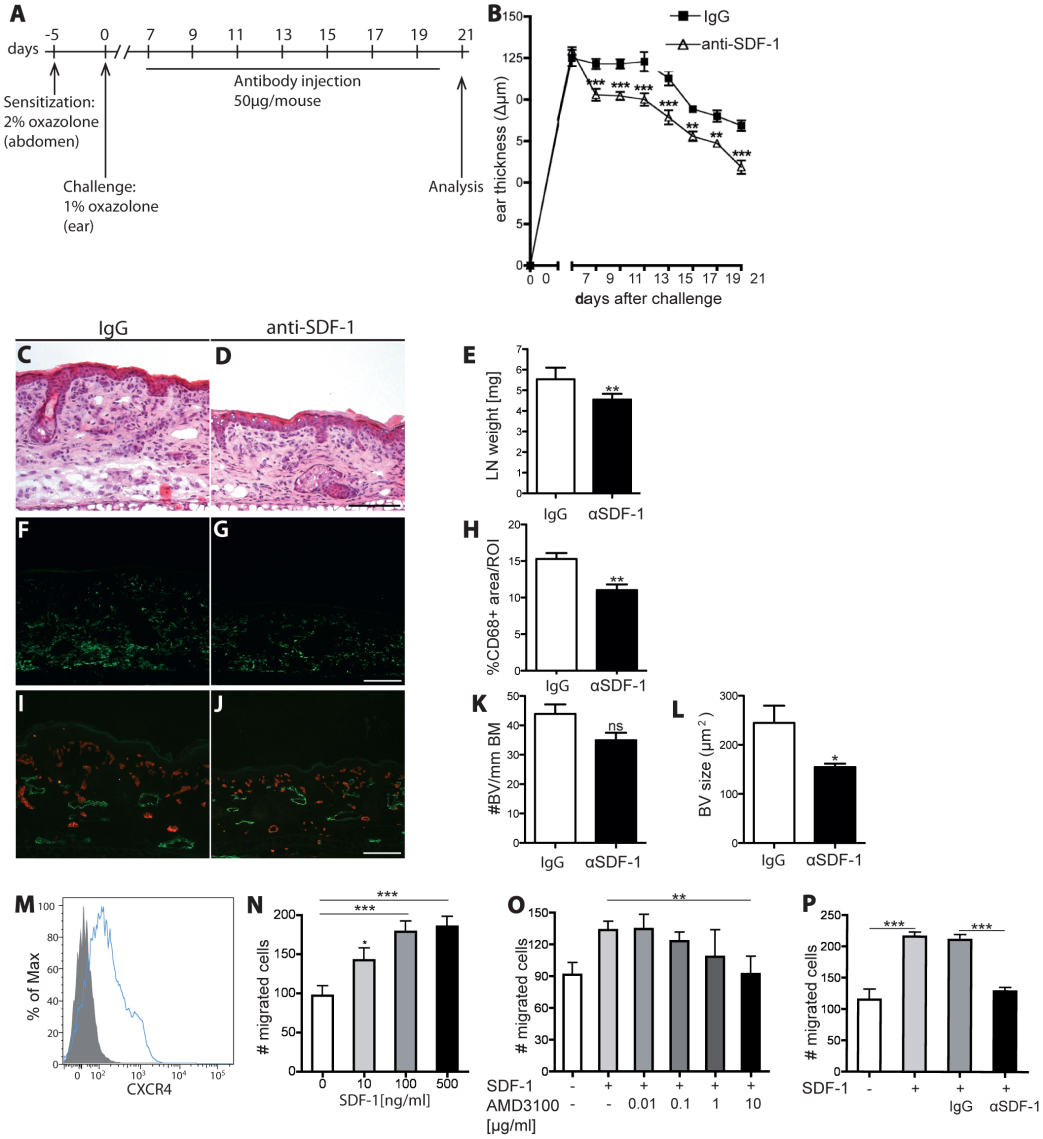
Inhibition of SDF-1 signaling alleviates chronic skin inflammation and inhibits leukocyte migration towards SDF. (A) On day -5, hemizygous K14 VEGF-A transgenic mice (n = 14) were sensitized with 2% oxazolone and challenged on day 0 with 1% oxazolone on the ears. Starting 7 days after challenge, mice received i.v injections of anti-SDF-1 antibody or isotype-matched IgG every second day. (B) Neutralization of SDF-1 (△) significantly reduced inflammatory ear swelling as compared with IgG-injected mice (□). Data represent mean ±SEM. (C-D) H&E stains of mouse ear sections at day 21 showed reduced edema and inflammatory cell infiltration in the anti-SDF-1 treated mice (D) in comparison to the IgG control group (C). One ear half is shown. Scale bar represents 100 μm. (E) In anti-SDF-1 treated mice, the weight of ear draining LNs was significantly reduced compared with controls. (F-H) Immunofluorescence staining for CD68 revealed a significant reduction in the percentage of area covered by macrophages in anti-SDF-1-treated animals as compared to IgG treatment. (I-L) Neutralization of SDF-1 decreased the size and numbers of blood vessels in the inflamed ear skin. (M) FACS analysis of CD11b^+^ splenocytes revealed a clear expression of CXCR4 on their surface. (N) SDF-1 promoted the chemotactic migration of CD11b^+^ splenocytes in vitro. (O-P) The chemotactic effect of SDF-1 was blocked by incubation with AMD3100 (O) or with an anti-SDF-1 antibody (P), but not with control IgG. Two independent experiments were performed. Data represent mean±SD. ^**^P<0.01; ^***^P<0.001.

Because blockade of CXCR4 inhibited chronic skin inflammation, we next investigated whether neutralization of the CXCR4 ligand SDF-1 might have comparable effects. To this end, we sensitized and challenged hemizygous K14-VEGF-A transgenic mice as described above. At 7 days after challenge, the mice were treated every second day by i.v. injection of an anti-SDF-1 antibody or of isotype-matched IgG. Mice were analyzed two days after the last antibody injection (day 21; Fig. 4A). A significant reduction of ear swelling was observed within 2 days of anti-SDF-1 antibody treatment, as compared with control IgG injection (day 9; −15.29%; P<0.001; Fig. 4B–D). The anti-inflammatory effect of the anti-SDF-1 antibody was reflected by the reduced weight of ear draining LNs at day 21 (−17.81%; P<0.01; Fig. 4E). Immunofluorescence analyses of inflamed ear skin showed a significant reduction of the macrophage-positive stained area (−27.90%, P<0.01) in anti-SDF-1-treated mice as compared to IgG-treated mice (Figure 4 F–H). SDF-1 neutralization also significantly reduced the size of blood vessels (−36.78%, P<0.05) and slightly reduced the number of blood vessels in comparison to IgG (−19.9%, P = 0.08) (Figure 4I–L).

To further investigate the role of the SDF-1/CXCR4 axis in leukocyte migration, we next performed an in vitro leukocyte chemotaxis assay using CD11b^+^ splenocytes. As shown by FACS analysis, these cells strongly express CXCR4 (Fig. 4M). We found that SDF-1 dose-dependently induced chemotaxis of CD11b^+^CXCR4^+^ splenocytes, with a minimal effective concentration of 100 ng/ml (Fig. 4N). The number of migrated cells was dose-dependently decreased when the cells were pre-incubated with AMD3100. At a concentration of 10 μg/ml, AMD3100 completely blocked the chemoattractant effect of SDF-1 (Fig. 4O). Addition of the anti-SDF-1 blocking antibody also significantly decreased the amount of migrated cells in response to SDF-1 when compared to control IgG (−39.3%; P<0.001; Fig. 4P).

### Blockade of the SDF-1/CXCR4 axis normalizes epidermal architecture and decreases inflammatory cell infiltration in inflamed skin

**Figure 5:**
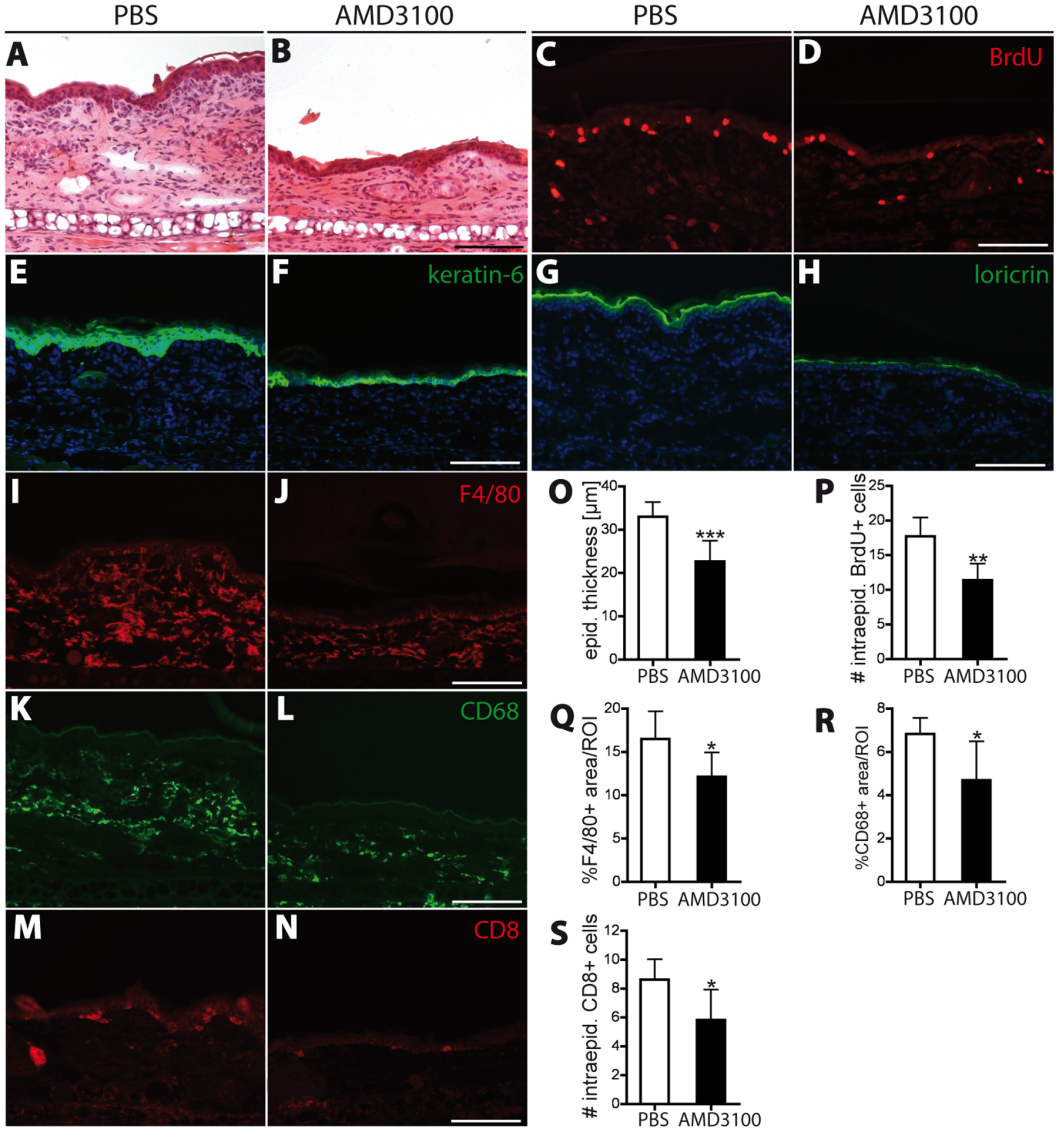
Inhibition of CXCR4 reduces inflammatory cell infiltration into the skin and normalizes epidermal architecture. (A–B) H&E stains of ear skin sections at day 21 showed that AMD3100 treatment reduced edema formation, epidermal thickening and inflammatory cell infiltration. (C–D) CXCR4 inhibition reduced the number of intraepidermal BrdU^+^ proliferating cells in the inflamed ear skin. (E–H) The hyperproliferation-associated keratin 6 and loricrin, a marker of terminal epidermal differentiation, were less broadly expressed in the epidermis of AMD3100-treated mice than in PBS-treated mice. (I–L) Immunofluorescence staining of the two macrophage markers F4/80 and CD68 revealed a significant reduction in the percentage of area covered by macrophages in AMD3100-treated mice compared to PBS treatment. (M–N) Inhibition of CXCR4 decreased the number of intraepidermal CD8^+^ T-cells in the inflamed ear skin. One ear half is shown. (O–P) Computer-assesed quantification of epidermal thickness (O), number of intraepidermal BrdU^+^ cells (P), the percentage of covered area by F4/80 (Q) and CD68 (R) postitive macrophages and the number of intraepidermal CD8^+^ cells (S). Scale bars represent 100 μm. Data represent mean ±SD. ^*^P<0.05; ^**^P<0.01; ^***^P<0.001.

Characteristic features of human psoriasis are marked thickening of the epidermis, due to increased proliferation of keratinocytes in the interfollicular epidermis, as well as abnormal terminal differentiation of keratinocytes [2]. At day 21 (after 14 days of treatment), control PBS-treated mice showed marked inflammatory dermal infiltrates and epidermal thickening (Fig. 5A), many numbers of BrdU-positive proliferating epidermal keratinocytes (Fig. 5C), and strong expression of the proliferation marker keratin 6 (K6; Fig. 5E) and the terminal differentiation marker loricrin (Fig. 5G). In contrast, AMD3100 treatment resulted in a reduction of epidermal thickness and of dermal inflammatory infiltrates (Fig. 5B, O). Also, the expression of keratin 6 and of loricrin was reduced (Fig. 5F, H), and the number of proliferating intraepidermal BrdU^+^ cells was significantly reduced (−36%; P<0.01; Fig. 5D, P).

Human psoriatic skin is also characterized by increased numbers of CD4^+^ T-cells, dendritic cells and macrophages within the dermis [2], [3], [33]. We next quantified the effect of CXCR4 blockade on the recruitment of macrophages into the inflamed ear skin, using two different macrophage markers, F4/80 and CD68 (Fig. 5I–L). The percentage of tissue area covered by macrophages was significantly reduced after AMD3100 treatment for both macrophage markers (by 27 and 31% respectively, P<0.05; Fig. 5Q-R). The infiltration of CD8^+^ T cells into the epidermis is a key feature of human psoriasis and of chronic skin inflammation in K14-VEGF-A transgenic mice [2], [9]. We found that the number of intraepidermal CD8^+^ cells was reduced by 33% (P<0.05) in the AMD3100-treated mice, as compared with PBS-injected mice (Fig.5 M–N, S). These results indicate that inhibition of the CXCR4 receptor inhibits recruitment of inflammatory cells into the inflamed skin.

To further corroborate these results and to study whether neutralization of SDF-1 might also lead to comparable effects, we next performed FACS analyses of distinct leukocyte subpopulations that were isolated from the inflamed ear skin of AMD3100 and PBS-treated mice, and of anti-SDF-1 and control-IgG-treated mice. All investigated inflammatory cell types (CD4^+^ and CD8^+^ T cells, dendritic cells, CXCR4^+^ dendritic cells, macrophages and CXCR4^+^ macrophages) were reduced after AMD3100 treatment and also after anti-SDF-1 treatment, compared to control treated mice (Fig. S1 in File S1).

### Blockade of CXCR4 reduces psoriasis-like skin inflammation induced by imiquimod

To investigate the role of the SDF-1/CXCR4 axis in a second mouse model of psoriasis, we used the imiquimod-induced psoriasis-like skin inflammation model [24], [25].

**Figure 6:**
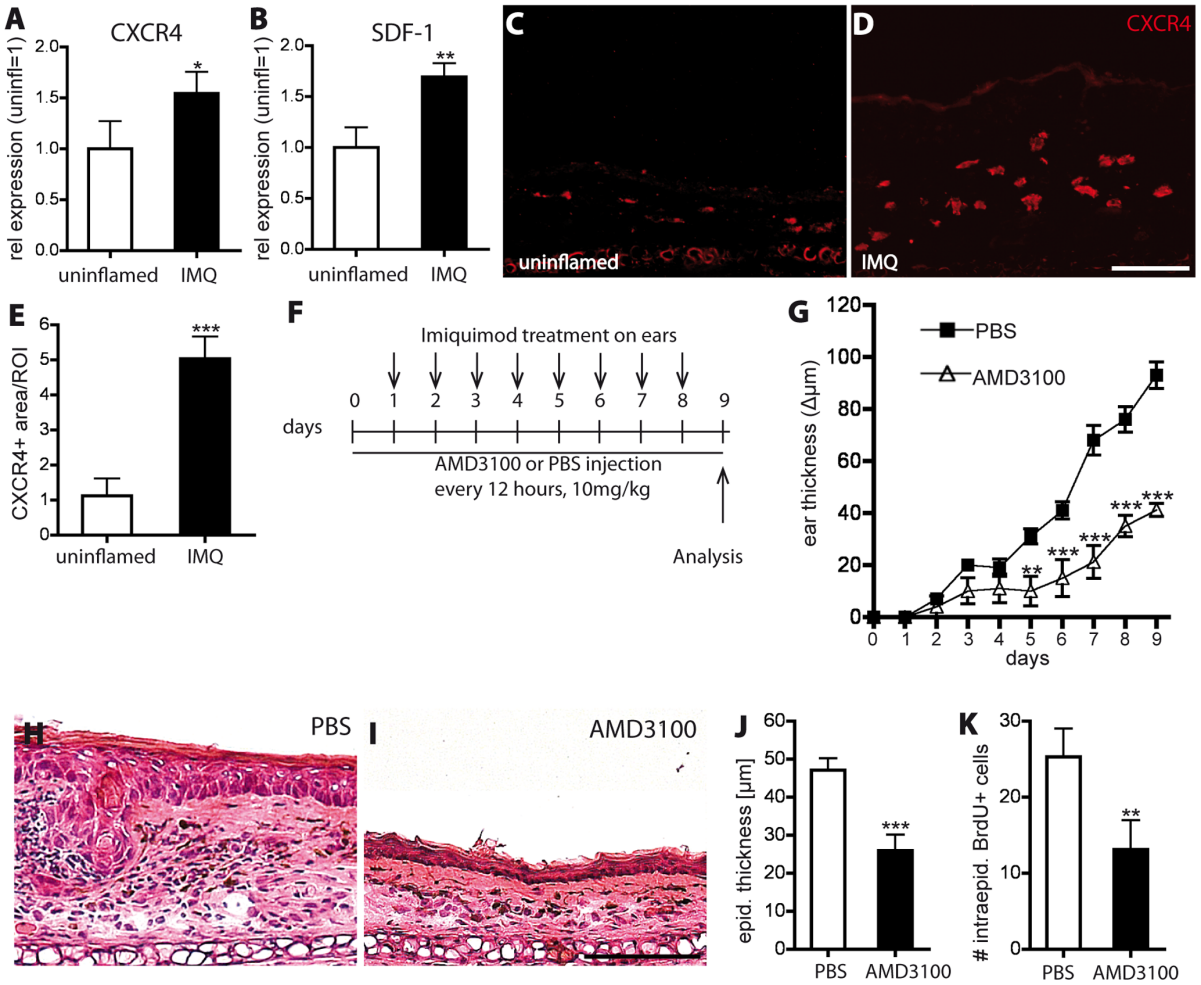
Inhibition of CXCR4 reduces imiquimod-induced skin inflammation. (A, B) Real-time RT-PCR analysis of extracts of IMQ-inflamed ear skin (8 consecutive days) and non-inflamed skin (n = 5 per group). The expression of CXCR4 and SDF-1 was significantly upregulated in IMQ-treated ear skin compared to uninflamed skin. (C–E) Immunofluorescence stains of ear skin for CXCR4 revealed a significantly increased CXCR4^+^ tissue area in IMQ-treated mice as compared with uninflamed skin. (F) Mice (n = 5 per group) received AMD3100 or PBS injections every 12 hours. 12 hours after the first injection, IMQ was applied topically, followed by daily applications for 8 days. (G) Treatment with AMD3100 (△) significantly reduced ear swelling as compared with PBS-treated controls (□). (H–J) H&E stains of ear skin sections showed that AMD3100 treatment (I) significantly reduced epidermal thickening compared to PBS-treated mice (H). Scale bar represents 100 μm. (K) CXCR4 inhibition significantly reduced the number of intraepidermal BrdU^+^ proliferating cells in the inflamed ear skin, as compared with control mice. Data represent mean±SD. ^*^P<0.05; ^**^P<0.01; ^***^P<0.001.

Real-time RT-PCR analyses of mouse ear skin from uninflamed and from imiquimod (IMQ)-treated mice (treatment for 8 consecutive days) revealed a significant increase in the expression of CXCR4 (1.5-fold, P<0.05) and of SDF-1 (1.7-fold, P<0.01) in the inflamed skin (Fig. 6A–B). CXCR7 was slightly downregulated in the IMQ-inflamed skin compared to uninflamed skin (data not shown). Immunofluorescence stainings demonstrated a higher number of CXCR4 expressing cells in IMQ-inflamed skin than in uninflamed skin (Fig. 6C–D). Quantitative image analyses revealed that the percentage of tissue area that stained positively for CXCR4 was significantly increased (347%, P<0.001) in the IMQ-inflamed ear skin as compared with uninflamed skin (Fig. 6E).

Next, C57BL/6 wild-type mice were treated with s.c. injections of AMD3100 every 12 hours. At 12 hours after the first injection, an imiquimod (IMQ)-containing cream (Aldara) was topically applied to the ear skin. IMQ was applied daily for 8 consecutive days and the mice were analyzed at day 9 (Fig. 6F). AMD3100 treatment significantly reduced ear thickness (Fig. 6G) and epidermal thickness (−47%; P<0.001; Fig. 6H–J) in the ears, as compared with control PBS-injected mice. The number of proliferating epidermal keratinocytes was significantly reduced after CXCR4 inhibition, compared to control treatment (−48%; P<0.01; Fig. 6K).

**Figure 7:**
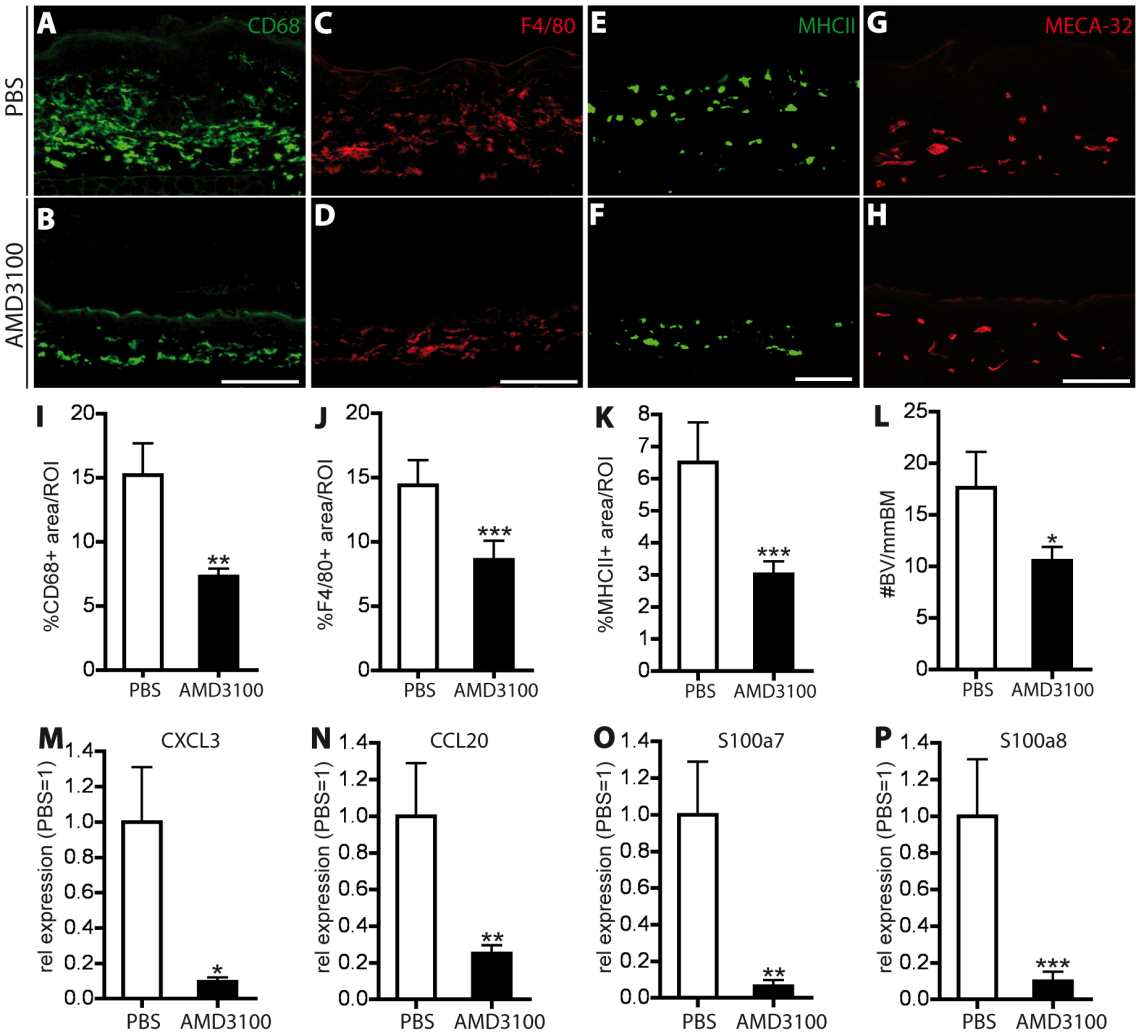
CXCR4 inhibition reduces inflammatory cell infiltration, angiogenesis and inflammatory marker expression in the imiquimod-induced skin inflammation. (A–H) Representative images of immunofluorescence stains for CD68^+^ (A, B) and F4/80^+^ (C, D) macrophages, MHCII^+^ antigen presenting cells (E, F) and MECA-32^+^ blood vessels (G, H) in the IMQ-inflamed ear skin of PBS and AMD3100-treated mice. One ear half is shown. Scale bars represent 100 μm. (I–K) Quantitative image analysis showed a significant reduction in the percentage of area covered by macrophages (I, J) and antigen presenting cells (K) in AMD3100-treated mice. (L) Inhibition of CXCR4 decreased the number of Meca-32^+^ blood vessels (BV). (M–P) Real-time RT-PCR analyses of RNA from whole ear skin extracts of imiquimod-inflamed mice showed a significant downregulation of CXCL3 (M), CCL20 (N), S100a7 (O) and S100a8 (P). Data represent mean ±SD. ^*^P<0.05; ^**^P<0.01; ^***^P<0.001.

Stainings for the macrophage markers CD68 and F4/80 revealed a significant reduction of the positively stained skin area (−42%; P<0.01 and −36.7%; P<0.001; Fig. 7A–D, I–J). Antigen presenting cells (MHCII^+^) were also significantly reduced upon AMD3100 treatment (−53.7%, P<0.001; Fig. 7E–F, K), whereas no significant change was observed in neutrophil infiltration (Gr-1^+^) between the two groups (data not shown).

As evaluated by staining for MECA-32, the number of cutaneous blood vessels was significantly decreased in AMD3100-treated mice, compared to control mice (−38%; P<0.05; Fig. 7G–H, L). The reduction of the cellular markers of inflammation after CXCR4 blockade was in agreement with the strongly decreased expression of the inflammatory markers CXCL3, CCL20, S100a7 and S100a8 (Fig. 7M-P).

## Discussion

In this study, we investigated the role of the SDF-1/CXCR4 axis in human psoriasis and in experimental chronic skin inflammation. Using a VEGF-A driven mouse model of cutaneous inflammation, we found that inhibition of CXCR4 by AMD3100, a specific CXCR4 antagonist, as well as neutralization of its ligand SDF-1 significantly improved the course of chronic skin inflammation. The anti-inflammatory effect of AMD3100 was further confirmed in a second, the imiquimod-induced model of skin inflammation.

Our findings of increased numbers of CXCR4^+^ cells in human psoriatic skin lesions are in line with the observed upregulation of CXCR4 and SDF-1 mRNA expression in psoriatic skin [21], [22] and with the detection of SDF-1 protein in psoriatic lesions [23]. It is of interest that increased numbers of CXCR4^+^ leukocytes have also been recently detected in other chronic inflammatory diseases, namely in the synovia of joints affected by rheumatoid arthritis and in the colonic tissue of patients with ulcerative colitis patients [34], [35]. Indeed, inhibition of CXCR4 was found to exert anti-inflammatory effects in experimental models of joint inflammation, colitis and allergic lung inflammation [28], [36]. Accordingly, increased expression of the CXCR4 ligand SDF-1 has also been reported in murine experimental colitis and collagen-induced arthritis, as well as in the synovial fluid of rheumatoid arthritis patients [34], [35], [37].

Importantly, in the present study, we found that both CXCR4 and SDF-1 were also upregulated in two independent experimental mouse models of chronic, psoriasis-like skin inflammation, namely the chronic skin inflammation induced in K14-VEGF-A transgenic mice [9], [10] and the cutaneous inflammation induced by topical treatment of mouse skin with imiquimod [24], [25]. In the VEGF-A transgenic model, dermal endothelial cells expressed increased levels of CXCR4, in line with the experimental upregulation of endothelial CXCR4 expression by VEGF-A in vitro [16]. The second receptor for SDF-1, CXCR7, has recently been described as a scavenger for SDF-1, and it has been suggested that ligand internalization and degradation by CXCR7 limits the availability of SDF-1 and thereby modulates the activity of CXCR4 [38]. It is therefore of interest that the CXCR7 expression was slightly decreased in the inflamed ear skin of both mouse models, suggesting that both upregulation of CXCR4 and downregulation of CXCR7 contribute to the pro-inflammatory effect of SDF-1 in chronically inflamed skin.

In the K14-VEGF-A transgenic mouse model, VEGF-A expression is strongly increased in the inflamed skin [39] and represents the major driver of inflammation-induced angiogenesis. VEGF-A binds to its high-affinity receptors VEGFR-1 and VEGFR-2, and we have previously found that inhibition of these two receptors by NVP-BAW2881, a VEGFR tyrosine-kinase inhibitor, exerts strong anti-inflammatory effects by reducing the number of infiltrating leukocytes and of blood vessels in the inflamed ear skin [11]. Furthermore, anti-VEGF-A antibody treatment strongly reduced skin inflammation, as well as the number and size of blood vessels, in a mouse model in which epidermal-specific deletion of c-Jun and JunB leads to a psoriasis-like skin inflammation [12]. These results indicate an important role of angiogenesis in the mediation of chronic skin inflammation. It is therefore of interest that we found CXCR4 to be specifically expressed by cultured dermal blood vascular endothelial cells but not lymphatic endothelial cells, and that CXCR4 was expressed by the small cutaneous blood vessels but not by lymphatic vessels in inflamed skin in situ. Importantly, our findings reveal, for the first time, that inhibition of CXCR4 reduces inflammation-induced angiogenesis, as shown in two independent mouse models, and they identify blood vessels as important targets of AMD3100. The involvement of CXCR4 in inflammatory angiogenesis is further supported by the reported activity of SDF-1 on in vitro tube formation, migration and sprouting of endothelial cells, all essential steps in the formation of new blood vessels, and on the in vivo angiogenic activity of SDF-1 in cutaneous and cornea assays [15], [16]. Even tough, inflammatory angiogenesis is likely to be an attractive target for the treatment of chronic skin inflammation, there is some evidence, that severe psoriasis is associated with a significant increase in cardiovascular diseases as compared to mild psoriasis and healthy individuals [40]. Thus, for systemic therapies with anti-angiogenic actions, high-risk cardiovascular patients would most likely have to be excluded.

AMD3100 has been described as specific CXCR4 antagonist [26]. To further investigate the potential role of the SDF-1/CXCR4 axis in inflammation, we performed a range of in vitro and in vivo studies using a neutralizing anti-SDF-1 antibody. These studies confirmed the important role of SDF-1-mediated CXCR4 activation in promoting chronic skin inflammation interaction and therefore validate the specific inhibition of the SDF-1/CXCR4 as a new therapeutic strategy to treat chronic inflammatory skin diseases.

Our results indicate that SDF-1 attracts CD11b^+^ splenocytes in a dose-dependent manner in vitro and that AMD3100 and anti-SDF-1 antibody block SDF-1 induced migration, in agreement with the reported chemoattractive activity of SDF-1 for mononuclear cells and different lymphocyte subpopulations in vitro and in vivo [41], [42]. Importantly, both AMD3100 and the SDF-1 blocking antibody also significantly reduced the number of different inflammatory cell subtypes in the inflamed ear skin in vivo. Together with the increased SDF-1 expression in the inflamed skin of K14-VEGF-A transgenic mice and of IMQ-treated mice, these data suggest that circulating CXCR4^+^ inflammatory cells are chemoattracted to the inflamed cutaneous tissue by SDF-1 and that this chemoattraction can be blocked by inhibiting the SDF-1/CXCR4 axis. Indeed, FACS analyses revealed reduced numbers of recruited CXCR4^+^ dendritic cells and CXCR4^+^ macrophages in the inflamed ear skin of K14-VEGF-A transgenic mice after either anti-SDF-1 or AMD3100 treatment.

It is of interest that inhibition of CXCR4 largely normalized all of the major pathological parameters of psoriatic skin lesions. Our findings indicate that blood vessels and several types of inflammatory cells are direct targets of SDF-1/CXCR4 axis inhibition, whereas epidermal normalization resulted most likely from the overall reduced inflammatory response. One has to keep in mind that psoriasis is a human-specific disease which is not naturally observed in animals, and that most experimental mouse models do not fully recapitulate all characteristics of the human disease, indicating that it might be advantageous to use more than just one mouse model for specific pathogenic studies of psoriasis. The K14-VEGF-A transgenic mouse model of chronic cutaneous inflammation shares several of the major characteristic histological features of human psoriatic skin. These include acanthosis of the epidermis, increased epidermal proliferation and abnormal differentiation, pathological angiogenesis and leukocyte infiltration with preferential location of CD8^+^ T cells in the epidermis and of CD4^+^ T cells in the dermis. Nevertheless, given the above mentioned concerns regarding mouse models of psoriasis, we investigated the role of the SDF-1/CXCR4 axis in a second mouse model of psoriasis, namely the IMQ-induced skin inflammation model. Importantly, these studies confirmed the findings of the VEGF-A transgenic mouse model, indicating that specific inhibition of the SDF-1/CXCR4 might represent a new therapeutic strategy to treat skin inflammation. In light of the potential adverse effects of systemic AMD3100 treatment, which include thrombocytopenia observed in HIV-infected patients [43], the development of topical anti-SDF-1/CXCR4 therapies might represent a more promising avenue for inhibiting chronic inflammatory skin disorders such as psoriasis.

## Supporting Information

File S1 Figure S1, Inhibition of the SDF-1/CXCR4 axis decreases the number of several types of inflammatory cells in chronically inflamed skin. Single-cell suspensions from inflamed ear skin were analyzed by FACS for the presence of different inflammatory cell populations. (A, B) Inflamed ear skin was analyzed for the presence of CD3^+^/CD4^+^ and CD3^+^/CD8^+^ T-cells. AMD3100 and anti-SDF-1 treatment significantly decreased the number of CD4^+^ cells, as compared with control mice. Compared to PBS-treated mice, AMD3100 further significantly decreased the number of CD8^+^ cells in the inflamed ear skin. Anti-SDF-1 showed the same tendency. (C, D) The number of dendritic cells (DC) was investigated by analyzing I-A/I-E^+^CD11c^+^ cells. Inhibition of the SDF-1/CXCR4 axis resulted in significantly reduced numbers of DC in the inflamed ear skin. Anti-SDF-1 treatment also significantly decreased the number of CXCR4^+^ DC. AMD3100 treatment showed the same trend. (E, F) Macrophages were assessed by evaluating the CD11b and F4/80 double positive population within live CD45^+^ leukocytes. AMD3100 significantly reduced the number of macrophages as well as of CXCR4^+^ macrophages in the inflamed ear skin. Anti-SDF-1 showed a tendency to reduce the number of these inflammatory cell populations and significantly reduced the number of CXCR4^+^ macrophages in the inflamed ear skin. n = 5 per group. Two independent experiments were performed. Data represent mean ±SD. ^*^P<0.05; ^***^P<0.001. ns, not significant.
(DOCX)
