# Supplementary material for: An Important Role of the SDF-1/CXCR4 Axis in Chronic Skin Inflammation
Source: PLoS One. 2014 Apr 2;9(4):e93665. doi: 10.1371/journal.pone.0093665 (PMC3973543; doi:10.1371/journal.pone.0093665)

**Materials and Methods S1**

**FACS analysis of cell suspensions from ears**

Ears were split into dorsal and ventral halves using forceps and the ear halves were transferred, dermal side down, into a plate containing RPMI-1640 media (Invitrogen) supplemented with 10% FBS and antibiotics at 37°C. The leukocyte populations were allowed to migrate over 12 hours from the ear halves into the media and were stained for FACS analysis using the same antibodies as described in the previous section for FACS analysis of LNs. Additionally, PerCP-labeled anti-mouse CD45 (BD Biosciences), FITC-labeled anti-mouse CD11b (Biolegend) and APC-labeled anti-mouse F4/80 (eBioscience) were used. The events were divided into CXCR4^+^ and CXCR4^-^ populations based upon a fluorescence minus one (FMO) control([1](#_ENREF_1)). To quantify total CD4, CD8, dendritic cell, CXCR4+ dendritic cell, macrophage and CXCR4+ macrophage numbers in ear samples, all migrated cells were analyzed.

1. Roederer, M. (2001) Spectral compensation for flow cytometry: visualization artifacts, limitations, and caveats. *Cytometry* **45**, 194-205


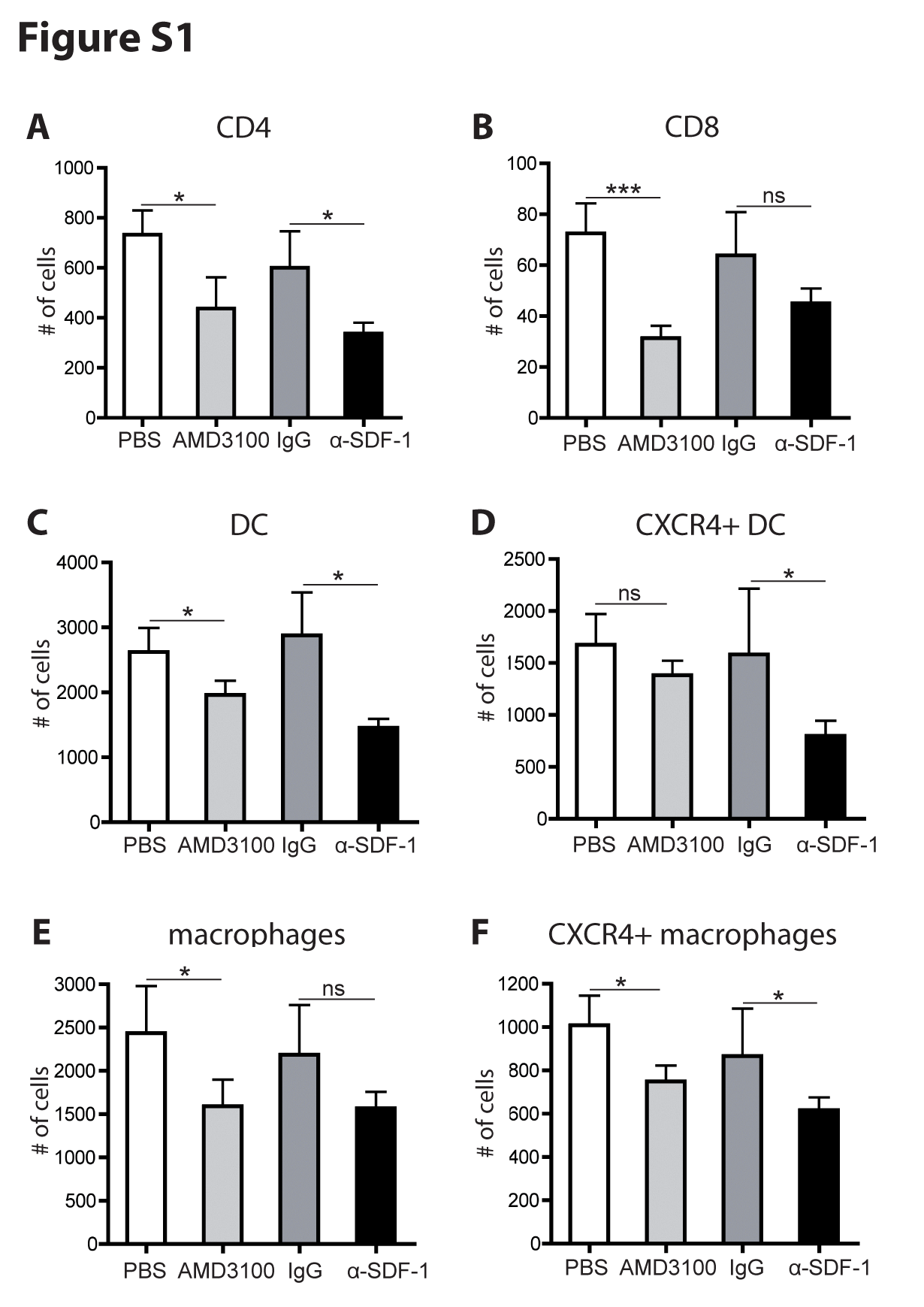

Supplement: File S1 — Figure S1, Inhibition of the SDF-1/CXCR4 axis decreases the number of several types of inflammatory cells in chronically inflamed skin. Single-cell suspensions from inflamed ear skin were analyzed by FACS for the presence of different inflammatory cell populations. (A, B) Inflamed ear skin was analyzed for the presence of CD3+/CD4+ and CD3+/CD8+ T-cells. AMD3100 and anti-SDF-1 treatment significantly decreased the number of CD4+ cells, as compared with control mice. Compared to PBS-treated mice, AMD3100 further significantly decreased the number of CD8+ cells in the inflamed ear skin. Anti-SDF-1 showed the same tendency. (C, D) The number of dendritic cells (DC) was investigated by analyzing I-A/I-E+CD11c+ cells. Inhibition of the SDF-1/CXCR4 axis resulted in significantly reduced numbers of DC in the inflamed ear skin. Anti-SDF-1 treatment also significantly decreased the number of CXCR4+ DC. AMD3100 treatment showed the same trend. (E, F) Macrophages were assessed by evaluating the CD11b and F4/80 double positive population within live CD45+ leukocytes. AMD3100 significantly reduced the number of macrophages as well as of CXCR4+ macrophages in the inflamed ear skin. Anti-SDF-1 showed a tendency to reduce the number of these inflammatory cell populations and significantly reduced the number of CXCR4+ macrophages in the inflamed ear skin. n = 5 per group. Two independent experiments were performed. Data represent mean ±SD. *P<0.05; ***P<0.001. ns, not significant. (DOCX) [file pone.0093665.s001.docx]
